# *N*-Ethyl-*N*-(4-methyl­phen­yl)benzene­sulfonamide

**DOI:** 10.1107/S1600536812000177

**Published:** 2012-01-11

**Authors:** Muhammad Akhyar Farrukh, Komal Faryal, Maymoona Mahboob, Fahim Ashraf Qureshi, Mehmet Akkurt

**Affiliations:** a Department of Chemistry, Government College University, Lahore 54000, Pakistan; b Department of Physics, Faculty of Sciences, Erciyes University, 38039 Kayseri, Turkey

## Abstract

The title compound, C_15_H_17_NO_2_S, is twisted at the S—N bond with a C—S—N—C torsion angle of 73.90 (14)°. The dihedral angle between the aromatic rings is 36.76 (11)°.

## Related literature

For related structures, see: Ahmad *et al.* (2011 ▶); Nirmala *et al.* (2011 ▶). For applications of sulfonamides, see: Faidallah *et al.* (2007 ▶); Gauss & Weinstein (1946 ▶); Korolkovas (1988 ▶); Laurence (2009 ▶).
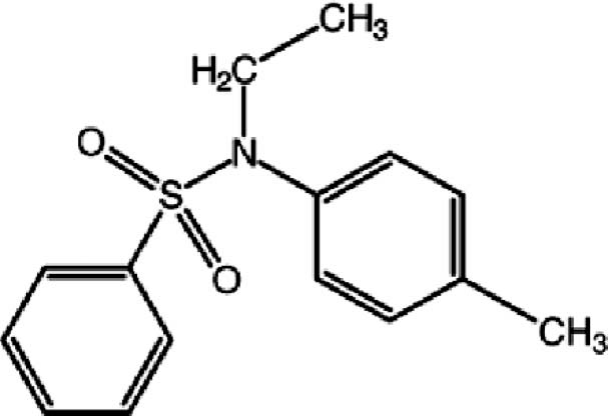


## Experimental

### 

#### Crystal data

C_15_H_17_NO_2_S
*M* *_r_* = 275.37
Orthorhombic, 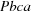

*a* = 15.6737 (5) Å
*b* = 8.2831 (2) Å
*c* = 22.3326 (7) Å
*V* = 2899.37 (15) Å^3^
*Z* = 8
Mo *K*α radiation
μ = 0.22 mm^−1^
*T* = 296 K
0.20 × 0.19 × 0.15 mm

#### Data collection

Bruker APEXII CCD diffractometer
26615 measured reflections
3592 independent reflections
2558 reflections with *I* > 2σ(*I*)
*R*_int_ = 0.024

#### Refinement

*R*[*F* ^2^ > 2σ(*F* ^2^)] = 0.044
*wR*(*F* ^2^) = 0.136
*S* = 1.02
3592 reflections
174 parameters
H-atom parameters constrained
Δρ_max_ = 0.21 e Å^−3^
Δρ_min_ = −0.37 e Å^−3^

### 

Data collection: *APEX2* (Bruker, 2007 ▶); cell refinement: *SAINT* (Bruker, 2007 ▶); data reduction: *SAINT*; program(s) used to solve structure: *SHELXS97* (Sheldrick, 2008 ▶); program(s) used to refine structure: *SHELXL97* (Sheldrick, 2008 ▶); molecular graphics: *ORTEP-3 for Windows* (Farrugia, 1997 ▶); software used to prepare material for publication: *WinGX* (Farrugia, 1999 ▶) and *PLATON* (Spek, 2009 ▶).

## Supplementary Material

Crystal structure: contains datablock(s) global, I. DOI: 10.1107/S1600536812000177/is5044sup1.cif

Structure factors: contains datablock(s) I. DOI: 10.1107/S1600536812000177/is5044Isup2.hkl

Supplementary material file. DOI: 10.1107/S1600536812000177/is5044Isup3.cml

Additional supplementary materials:  crystallographic information; 3D view; checkCIF report

## Figures and Tables

**Fig. 1.**
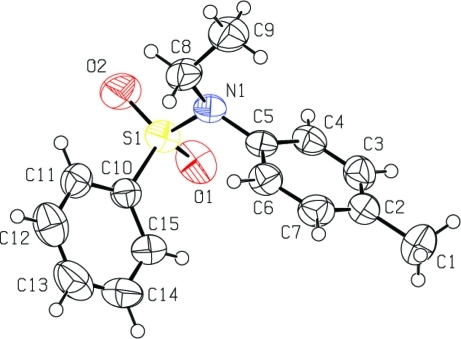
The molecular structure of the title compound, showing the atom-labelling scheme and displacement ellipsoids at the 50% probability level.

**Fig. 2.**
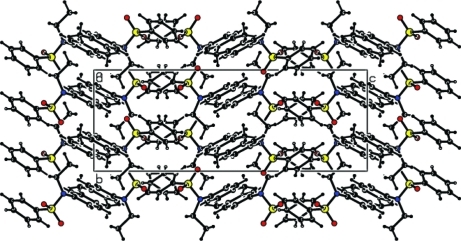
A packing diagram of the title compound, viewed down the a axis in the unit cell.

**Fig. 3.**
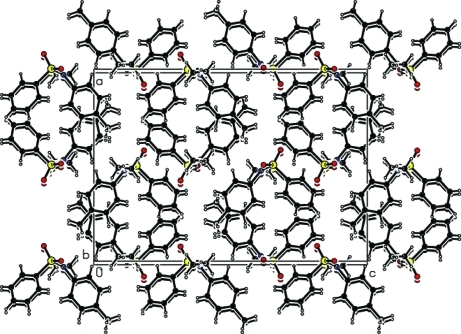
A packing diagram of the title compound, viewed down the b axis in the unit cell.

## supplementary crystallographic information

### Comment

Sulfonamides commonly named as Sulfa drugs are the medicines capable of controlling the bacterial infections (Laurence, 2009). The phenolic azo- dyes derived from the sulfonamides have the therapeutic potentialities and special mode of action against the acute bacterial infections (Korolkovas, 1988). Some benzenesulfonamide are evaluated for their *in vitro* antitumor activity (Faidallah *et al.*, 2007). Hemorrhagic colitis (i.e. swollen of Colon and diarrhea) can be the direct result of the toxic effect of the ingested sulfonamide but with the withdrawal of the sulfonamides from the body, the symptoms subsided and body returns to its normal activity (Gauss & Weinstein, 1946) As part of our ongoing studies of the effect of substitutions on the structures of *N*-(aryl)-arylsulfonamides (Ahmad *et al.*, 2011), we synthesized the title compound, (I), and report herein its crystal structure.

As shown in Fig. 1, the title molecule is twisted at the S—N bond with the C10—S1—N1—C5 torsion angle of 73.90 (14)°, compared to the values of 80.2 (3)° (molecule 1) and -79.4 (3)° (molecule 2) in *N*-ethyl-4-methyl-*N*-(3-methylphenyl)benzenesulfonamide (II) (Ahmad *et al.*, 2011), -58.4 (2) and -48.3 (2)° (molecule 1) and -75.7 (3)° (molecule 2), in the two molecules of 2,4-dimethyl-*N*-(4-methylphenyl)benzenesulfonamide (III) (Nirmala *et al.*, 2011). The phenyl and benzene rings in (I) are tilted relative to each other by 36.76 (11)°, compared to the values of 35.3 (2)° (molecule 1) and 42.5 (2)° (molecule 2) in (II), and 72.0 (1)° (molecule 1) and 78.3 (1)° (molecule 2) in (III). No classical hydrogen bonds are observed in the crystal structure. The crystal packing of (I) is shown in Figs. 2 & 3 down the *a* and *b* axes, respectively.

### Experimental

5 m*M* of *p*-toluidine was dissolved in 20 ml of distilled water then 5 m*M* of ethyl iodide was added. The reaction mixture was stirred properly and 5 m*M* of benzenesulfonyl chloride was added. The mixture was stirred for about 1–2 h and the pH was maintained 8–10 using 3% Na_2_CO_3_ solution. The reaction was monitored by TLC. The product obtained was filtered and the precipitate was washed with distilled water, dried and recrystallized using methanol.

### Refinement

All H atoms were geometrically positioned and refined using a riding model with C—H = 0.93–0.97 Å. The *U*_iso_(H) values were constrained to be 1.5*U*_eq_(methyl-C) or 1.2*U*_eq_(other C atoms). In the final refinement one low angle reflection, 0 0 2, evidently effected by the beam stop was omitted.

### Crystal data

**Table:**

C_15_H_17_NO_2_S	*F*(000) = 1168
*M**_r_* = 275.37	*D*_x_ = 1.262 Mg m^−^^3^
Orthorhombic, *P**b**c**a*	Mo *K*α radiation, λ = 0.71073 Å
Hall symbol: -P 2ac 2ab	Cell parameters from 8605 reflections
*a* = 15.6737 (5) Å	θ = 2.2–27.8°
*b* = 8.2831 (2) Å	µ = 0.22 mm^−^^1^
*c* = 22.3326 (7) Å	*T* = 296 K
*V* = 2899.37 (15) Å^3^	Prism, colourless
*Z* = 8	0.20 × 0.19 × 0.15 mm

### Data collection

**Table:**

Bruker APEXII CCD diffractometer	2558 reflections with *I* > 2σ(*I*)
Radiation source: sealed tube	*R*_int_ = 0.024
graphite	θ_max_ = 28.3°, θ_min_ = 2.6°
φ and ω scans	*h* = −20→20
26615 measured reflections	*k* = −11→10
3592 independent reflections	*l* = −29→28

### Refinement

**Table:**

Refinement on *F*^2^	Primary atom site location: structure-invariant direct methods
Least-squares matrix: full	Secondary atom site location: difference Fourier map
*R*[*F*^2^ > 2σ(*F*^2^)] = 0.044	Hydrogen site location: inferred from neighbouring sites
*wR*(*F*^2^) = 0.136	H-atom parameters constrained
*S* = 1.02	*w* = 1/[σ^2^(*F*_o_^2^) + (0.0634*P*)^2^ + 0.8829*P*] where *P* = (*F*_o_^2^ + 2*F*_c_^2^)/3
3592 reflections	(Δ/σ)_max_ = 0.001
174 parameters	Δρ_max_ = 0.21 e Å^−^^3^
0 restraints	Δρ_min_ = −0.37 e Å^−^^3^

### Special details

**Table:**

Geometry. Bond distances, angles *etc*. have been calculated using the rounded fractional coordinates. All su's are estimated from the variances of the (full) variance-covariance matrix. The cell e.s.d.'s are taken into account in the estimation of distances, angles and torsion angles
Refinement. Refinement on *F*^2^ for ALL reflections except those flagged by the user for potential systematic errors. Weighted *R*-factors *wR* and all goodnesses of fit *S* are based on *F*^2^, conventional *R*-factors *R* are based on *F*, with *F* set to zero for negative *F*^2^. The observed criterion of *F*^2^ > σ(*F*^2^) is used only for calculating -*R*-factor-obs *etc*. and is not relevant to the choice of reflections for refinement. *R*-factors based on *F*^2^ are statistically about twice as large as those based on *F*, and *R*-factors based on ALL data will be even larger.

### Fractional atomic coordinates and isotropic or equivalent isotropic displacement parameters (Å^2^)

**Table:**

	*x*	*y*	*z*	*U*_iso_*/*U*_eq_	
S1	0.99560 (3)	0.12782 (6)	0.34475 (2)	0.0552 (2)	
O1	0.99210 (11)	−0.01640 (16)	0.37951 (7)	0.0776 (6)	
O2	1.07355 (8)	0.1734 (2)	0.31684 (8)	0.0823 (6)	
N1	0.96703 (9)	0.27682 (16)	0.38880 (7)	0.0493 (4)	
C1	0.69051 (18)	0.1542 (4)	0.55144 (12)	0.1005 (11)	
C2	0.76285 (14)	0.1879 (3)	0.50850 (9)	0.0639 (7)	
C3	0.84503 (14)	0.1397 (2)	0.52122 (9)	0.0638 (7)	
C4	0.91137 (12)	0.1692 (2)	0.48250 (8)	0.0535 (6)	
C5	0.89614 (10)	0.24632 (18)	0.42854 (8)	0.0453 (5)	
C6	0.81402 (11)	0.2959 (2)	0.41530 (9)	0.0568 (6)	
C7	0.74880 (12)	0.2675 (3)	0.45512 (9)	0.0641 (7)	
C8	0.97391 (15)	0.4412 (2)	0.36295 (9)	0.0634 (6)	
C9	0.97675 (16)	0.5671 (2)	0.41062 (11)	0.0748 (8)	
C10	0.91654 (11)	0.11661 (19)	0.28917 (8)	0.0486 (5)	
C11	0.92329 (12)	0.2099 (2)	0.23824 (8)	0.0581 (6)	
C12	0.85857 (16)	0.2085 (3)	0.19712 (10)	0.0785 (9)	
C13	0.78769 (16)	0.1158 (4)	0.20632 (12)	0.0901 (10)	
C14	0.78161 (17)	0.0215 (4)	0.25675 (13)	0.0975 (10)	
C15	0.84602 (15)	0.0210 (3)	0.29854 (10)	0.0759 (8)	
H1A	0.64400	0.22620	0.54320	0.1510*	
H1B	0.70980	0.17040	0.59180	0.1510*	
H1C	0.67180	0.04460	0.54660	0.1510*	
H3	0.85590	0.08580	0.55700	0.0770*	
H4	0.96650	0.13750	0.49250	0.0640*	
H6	0.80290	0.34860	0.37940	0.0680*	
H7	0.69400	0.30280	0.44590	0.0770*	
H8A	0.92540	0.46110	0.33700	0.0760*	
H8B	1.02520	0.44810	0.33880	0.0760*	
H9A	0.92480	0.56410	0.43330	0.1120*	
H9B	0.98310	0.67160	0.39260	0.1120*	
H9C	1.02420	0.54670	0.43670	0.1120*	
H11	0.97140	0.27330	0.23190	0.0700*	
H12	0.86290	0.27110	0.16270	0.0940*	
H13	0.74360	0.11660	0.17850	0.1080*	
H14	0.73360	−0.04250	0.26270	0.1170*	
H15	0.84200	−0.04300	0.33260	0.0910*	

### Atomic displacement parameters (Å^2^)

**Table:**

	*U*^11^	*U*^22^	*U*^33^	*U*^12^	*U*^13^	*U*^23^
S1	0.0479 (3)	0.0516 (3)	0.0661 (3)	0.0083 (2)	−0.0039 (2)	−0.0070 (2)
O1	0.1042 (12)	0.0495 (7)	0.0790 (10)	0.0247 (7)	−0.0153 (8)	−0.0011 (7)
O2	0.0418 (7)	0.1055 (12)	0.0996 (11)	0.0043 (7)	0.0069 (8)	−0.0201 (10)
N1	0.0498 (8)	0.0415 (7)	0.0567 (8)	−0.0032 (6)	−0.0028 (7)	−0.0023 (6)
C1	0.0921 (19)	0.125 (2)	0.0843 (17)	−0.0261 (16)	0.0305 (15)	−0.0100 (16)
C2	0.0706 (13)	0.0640 (11)	0.0571 (11)	−0.0154 (9)	0.0070 (10)	−0.0114 (9)
C3	0.0817 (14)	0.0591 (11)	0.0505 (10)	−0.0076 (9)	−0.0047 (10)	0.0046 (8)
C4	0.0569 (10)	0.0487 (9)	0.0550 (10)	−0.0009 (7)	−0.0140 (8)	0.0004 (8)
C5	0.0460 (8)	0.0370 (7)	0.0528 (9)	−0.0030 (6)	−0.0055 (7)	−0.0031 (6)
C6	0.0520 (10)	0.0608 (10)	0.0575 (10)	0.0024 (8)	−0.0085 (8)	0.0065 (8)
C7	0.0475 (9)	0.0712 (12)	0.0736 (13)	0.0002 (9)	−0.0037 (9)	−0.0068 (10)
C8	0.0719 (12)	0.0460 (9)	0.0724 (12)	−0.0116 (8)	0.0050 (10)	0.0038 (9)
C9	0.0884 (15)	0.0459 (10)	0.0902 (15)	−0.0077 (10)	0.0048 (13)	−0.0074 (10)
C10	0.0463 (8)	0.0466 (8)	0.0529 (9)	0.0011 (7)	0.0031 (7)	−0.0073 (7)
C11	0.0565 (10)	0.0588 (10)	0.0591 (11)	0.0046 (8)	0.0087 (9)	−0.0019 (9)
C12	0.0863 (16)	0.0874 (16)	0.0619 (13)	0.0235 (13)	−0.0069 (12)	−0.0038 (11)
C13	0.0702 (15)	0.125 (2)	0.0750 (16)	0.0118 (15)	−0.0198 (12)	−0.0314 (16)
C14	0.0711 (15)	0.128 (2)	0.0934 (18)	−0.0409 (15)	−0.0007 (13)	−0.0304 (18)
C15	0.0788 (14)	0.0820 (14)	0.0668 (12)	−0.0338 (12)	0.0015 (11)	−0.0052 (11)

### Geometric parameters (Å, °)

**Table:**

S1—O1	1.4257 (15)	C14—C15	1.375 (4)
S1—O2	1.4226 (15)	C1—H1A	0.9600
S1—N1	1.6406 (15)	C1—H1B	0.9600
S1—C10	1.7564 (18)	C1—H1C	0.9600
N1—C5	1.444 (2)	C3—H3	0.9300
N1—C8	1.483 (2)	C4—H4	0.9300
C1—C2	1.511 (4)	C6—H6	0.9300
C2—C3	1.378 (3)	C7—H7	0.9300
C2—C7	1.380 (3)	C8—H8A	0.9700
C3—C4	1.374 (3)	C8—H8B	0.9700
C4—C5	1.385 (2)	C9—H9A	0.9600
C5—C6	1.383 (2)	C9—H9B	0.9600
C6—C7	1.375 (3)	C9—H9C	0.9600
C8—C9	1.491 (3)	C11—H11	0.9300
C10—C11	1.379 (2)	C12—H12	0.9300
C10—C15	1.376 (3)	C13—H13	0.9300
C11—C12	1.368 (3)	C14—H14	0.9300
C12—C13	1.366 (4)	C15—H15	0.9300
C13—C14	1.374 (4)		
			
O1—S1—O2	119.60 (10)	H1A—C1—H1C	110.00
O1—S1—N1	107.06 (8)	H1B—C1—H1C	110.00
O1—S1—C10	108.26 (9)	C2—C3—H3	119.00
O2—S1—N1	107.31 (9)	C4—C3—H3	119.00
O2—S1—C10	108.08 (9)	C3—C4—H4	120.00
N1—S1—C10	105.72 (8)	C5—C4—H4	120.00
S1—N1—C5	116.54 (11)	C5—C6—H6	120.00
S1—N1—C8	115.94 (12)	C7—C6—H6	120.00
C5—N1—C8	117.12 (14)	C2—C7—H7	119.00
C1—C2—C3	121.1 (2)	C6—C7—H7	119.00
C1—C2—C7	121.1 (2)	N1—C8—H8A	109.00
C3—C2—C7	117.75 (19)	N1—C8—H8B	109.00
C2—C3—C4	121.73 (18)	C9—C8—H8A	109.00
C3—C4—C5	119.95 (17)	C9—C8—H8B	109.00
N1—C5—C4	118.87 (15)	H8A—C8—H8B	108.00
N1—C5—C6	122.18 (16)	C8—C9—H9A	109.00
C4—C5—C6	118.91 (16)	C8—C9—H9B	110.00
C5—C6—C7	120.18 (18)	C8—C9—H9C	109.00
C2—C7—C6	121.45 (18)	H9A—C9—H9B	109.00
N1—C8—C9	111.50 (16)	H9A—C9—H9C	109.00
S1—C10—C11	119.94 (13)	H9B—C9—H9C	109.00
S1—C10—C15	119.33 (15)	C10—C11—H11	120.00
C11—C10—C15	120.64 (18)	C12—C11—H11	120.00
C10—C11—C12	119.46 (18)	C11—C12—H12	120.00
C11—C12—C13	120.5 (2)	C13—C12—H12	120.00
C12—C13—C14	120.0 (2)	C12—C13—H13	120.00
C13—C14—C15	120.5 (3)	C14—C13—H13	120.00
C10—C15—C14	119.0 (2)	C13—C14—H14	120.00
C2—C1—H1A	109.00	C15—C14—H14	120.00
C2—C1—H1B	109.00	C10—C15—H15	120.00
C2—C1—H1C	109.00	C14—C15—H15	121.00
H1A—C1—H1B	109.00		
			
O1—S1—N1—C5	−41.37 (15)	C1—C2—C3—C4	179.8 (2)
O2—S1—N1—C5	−170.93 (13)	C7—C2—C3—C4	0.1 (3)
C10—S1—N1—C5	73.90 (14)	C3—C2—C7—C6	1.0 (3)
O1—S1—N1—C8	174.57 (14)	C1—C2—C7—C6	−178.8 (2)
O2—S1—N1—C8	45.02 (16)	C2—C3—C4—C5	−1.3 (3)
C10—S1—N1—C8	−70.16 (15)	C3—C4—C5—N1	179.61 (15)
N1—S1—C10—C11	87.16 (15)	C3—C4—C5—C6	1.5 (2)
O1—S1—C10—C11	−158.39 (14)	C4—C5—C6—C7	−0.5 (3)
O2—S1—C10—C11	−27.48 (17)	N1—C5—C6—C7	−178.50 (17)
N1—S1—C10—C15	−89.31 (17)	C5—C6—C7—C2	−0.8 (3)
O1—S1—C10—C15	25.14 (19)	S1—C10—C11—C12	−175.60 (16)
O2—S1—C10—C15	156.05 (17)	C15—C10—C11—C12	0.8 (3)
C5—N1—C8—C9	56.2 (2)	S1—C10—C15—C14	175.5 (2)
S1—N1—C5—C4	83.55 (17)	C11—C10—C15—C14	−0.9 (3)
C8—N1—C5—C4	−132.82 (17)	C10—C11—C12—C13	0.1 (3)
S1—N1—C5—C6	−98.45 (17)	C11—C12—C13—C14	−0.9 (4)
C8—N1—C5—C6	45.2 (2)	C12—C13—C14—C15	0.8 (5)
S1—N1—C8—C9	−159.94 (15)	C13—C14—C15—C10	0.2 (4)

### Hydrogen-bond geometry (Å, °)

**Table:**

*D*—H···*A*	*D*—H	H···*A*	*D*···*A*	*D*—H···*A*
C8—H8B···O2	0.97	2.45	2.902 (2)	108
C15—H15···O1	0.93	2.58	2.934 (3)	103
